# Inbreeding Depression under Drought Stress in the Rare Endemic *Echium wildpretii* (Boraginaceae) on Tenerife, Canary Islands

**DOI:** 10.1371/journal.pone.0047415

**Published:** 2012-10-24

**Authors:** Janosch Sedlacek, Bernhard Schmid, Diethart Matthies, Matthias Albrecht

**Affiliations:** 1 Institute of Evolutionary Biology and Environmental Studies, University of Zurich, Zurich, Switzerland; 2 Department of Ecology, Philipps-University Marburg, Marburg, Germany; 3 Biodiversity and Agricultural Landscapes, Research Station Agroscope Reckenholz-Tanikon, Zurich, Switzerland; University of Northampton, United Kingdom

## Abstract

How climate-change induced environmental stress may alter the effects of inbreeding in patchy populations of rare species is poorly understood. We investigated the fitness of progeny from experimental self- and cross-pollinations in eight populations of different size of *Echium wildpretii*, a rare endemic plant of the arid subalpine zone of the Canarian island of Tenerife. As control treatments we used open pollination and autonomous selfing. The seed set of open-pollinated flowers was 55% higher than that of autonomously selfed flowers, showing the importance of animal pollination for reproductive success. The seed set, seed mass and germination rate of seedlings of hand-selfed flowers was similar to that of hand-crossed flowers, indicating weak inbreeding depression (seed set –4.4%, seed mass –4.1%, germination –7.3%). Similarly, under normal watering there were no significant effects of inbreeding on seedling survival (–3.0%). However, under low watering of seedlings inbreeding depression was high (survival –50.2%). Seed set of open- and hand-outcrossed-pollinated flowers was higher in large than in small populations, possibly due to more frequent biparental inbreeding in the latter. However, later measures of progeny fitness were not significantly influenced by population size. We predict that increasing drought duration and frequency due to climate change and reductions of population sizes may increase inbreeding depression in this charismatic plant species and thus threaten its future survival in the longer term.

## Introduction

Island ecosystems are characterized by high levels of endemism [1], [2] and harbour much of the world’s biodiversity [3]. However, island endemics are highly vulnerable to recent, rapid anthropogenic impacts, such as climate change, habitat modification, and the invasion of alien species [4]–[6]. Such human-induced changes can cause or increase pollen limitation during plant reproduction [7], [8], particularly in rare species [9]–[11]. In animal-pollinated plants reproductive success can be limited by both quantity and quality of deposited pollen [12], [13]. Anthropogenic reductions of the size of plant populations can result in increased selfing or pollen transfer between close relatives (biparental inbreeding; [10], [14]–[18]). Inbreeding in turn may reduce seed set and offspring fitness because of self-incompatibility or increased homozygosity, thus resulting in inbreeding depression (e.g. [15], [19]).

Theoretical and empirical work suggests that inbreeding depression may be stronger under harsh and stressful environmental conditions than under benign conditions (e.g. [20]–[22]). Global climate change will result in the deterioration of the environmental conditions for many extant populations and levels of inbreeding depression may thus increase in populations prone to inbreeding [23]. This may be particularly true for endemic species that have frequently narrow ecological niches, small distributional ranges, and occur in patchy populations [22]–[25]. Hence, climate change and small population size may put endemic and endangered species in double jeopardy [23], [26]. We tested the combined effects of inbreeding and drought on a rare, charismatic and endemic plant of the Canarian island of Tenerife, *Echium wildpretii,* as a model species. Because the habitats of *E. wildpretii* have been increasingly affected by more frequent and longer summer droughts over the last decades ([27], [28], and references therein), we tested for inbreeding depression of selfed offspring both under conditions of sufficient water availability and drought. In particular, we addressed the following questions: (1) Does selfing result in inbreeding depression for seed set, seed mass, germination, seedling performance and seedling survival? (2) Does drought stress aggravate inbreeding depression for seedling performance and seedling survival? (3) Does the level of inbreeding depression vary with population size and does population size interact with drought stress jointly affecting inbreeding?

## Materials and Methods

### Study Species and Study Area

The giant rosette plant *Echium wildpretii* [H. Pearson ex Hook.f.] (Boraginaceae) is endemic to the Canary Islands, where it is represented by two subspecies. The pink-flowering *Echium wildpretii* ssp. *trichosyphon* [(Svent.) Bramwell] is endemic to La Palma, while the red-flowering Teide bugloss *Echium wildpretii* ssp. *wildpretii* [H. Pearson ex Hook.f.] (hereafter *Echium wildpretii*) is confined to the sub-alpine zone of mount Teide on Tenerife and a few small areas on La Palma [29], [30]. *Echium wildpretii* is a self-compatible, monocarpic plant that grows as a rosette for 5–10 years before producing a single inflorescence [31]. The columnar inflorescence grows up to 3 m tall and has a basal diameter of 10–70 cm, tapering towards the top [31], [32]. Cymes, each comprising four (top of inflorescence) to 30 flowers (base of inflorescence) coil up along the column. A maximum of four nutlets can develop from the four ovules in each flower [33]. Flowers are protandrous and open for 2 to 3 days [32]. During the female phase the split carpel is taller than the anthers [32]. The flowers open successively from the inside to the outside of a cyme. The total flowering time of a plant is 3–5 weeks. Flowers of *E. wildpretii* are visited by native insect species (mainly bees), honey bees, passerine birds and lizards [34].

**Figure 1:**
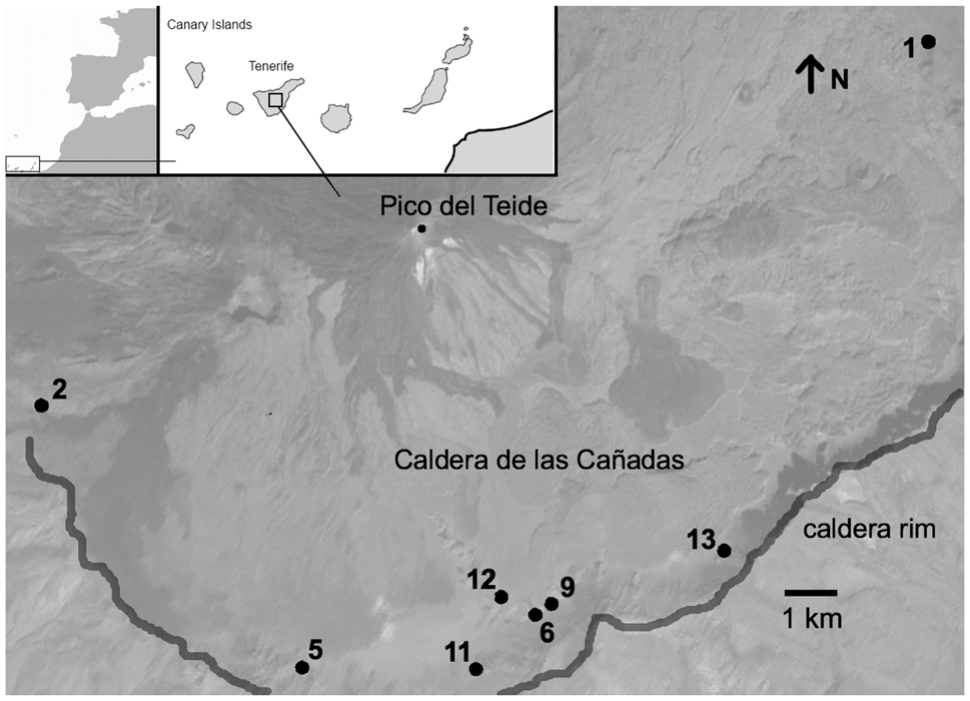
Study populations. Satellite image showing the location of the eight study populations (1 to 8) of *Echium wildpretii* ssp. *wildpretii* (Boraginaceae) in the caldera “Las Cañadas” of Mount Teide on Tenerife, Canary Islands. For detailed information on populations see Supporting Information 1.

*Echium wildpretii* is considered to be rare [35], but can be locally abundant. Most populations occur in the Mount Teide National Park inside the caldera “Las Cañadas” (2000–2300 m above sea level) that surrounds the top of Mount Teide on three sides (Fig. 1). The total size of populations (including seedlings) ranges from less than 10 to more than 3000 individuals. We selected eight populations ranging from 154–3380 individuals (mean: 1077±451) and 22–483 flowering individuals (mean: 126±55) inside the caldera (Fig. 1). Populations were at least 100 m apart from each other and grew from 2060–2200 m altitude.

The study area is characterized by a desert-like climate with a mean temperature of 10°C and strong daily temperature variation. Mean yearly precipitation is 440 mm with highest rainfall from November to March [36]. Few other plant species co-occur with *E. wildpretii*, and overall vegetation cover is low (35±5%, based on 64 10×10 m plots). The two Canary Island endemics *Pterocephalus lasiospermus* (Dipsacaceae) and *Spartocytisus supranubius* (Fabaceae) are the most abundant plants [31].

### Pollination Treatments

From 10–25 May 2009 we selected eight cymes in the centre of the inflorescence of eight flowering plants in each of the eight study populations. Two flowers in each of four randomly selected cymes per plant were marked and served as controls with open access to pollinators (open-pollinated, OP). The other four cymes were bagged with nylon mesh cloth (1 mm mesh size) to exclude pollinators. Approximately one week after bagging, two flowers in the female phase of each of the four cymes were randomly assigned to each of three pollination treatments (resulting in 8 flowers per plant for each pollination treatment): hand-crossing (HC), hand-selfing (HS), and autonomous selfing (AS). The flowers assigned to the two hand-pollination treatments (HC and HS) were emasculated with forceps before treatments were applied. For the hand-pollination treatments on a single plant c. 30 anthers from flowers of the pollen donor plant were removed with tweezers, stored in Petri dishes and applied within one hour after collection. For HC, pollen from the most distant of the eight flowering plants of the same population and, for HS, pollen from randomly selected flowers of the same plant was used. Flowers were hand-pollinated by rubbing pollen onto the lobes of the stigmata with a toothpick that was covered with parafilm [37]. With this method each stigma received on average 16.9±3.0 pollen grains (n = 30). The hand-pollinated flowers and flowers assigned to the AS treatment were marked and bagged again. All fruits that developed from the 1940 flowers used in the pollination experiment were collected from 12–16 July 2009, when seeds were ripe but not yet released from fruits. The number of seeds per fruit was counted, and seeds were weighed individually to determine seed mass. Permission to conduct research in Parque Nacional del Teide was issued by the park administration (Dr. Angel Bañares Baudet). No further permits were required for the described field studies.

### Germination Experiment

For germination, the seeds were put into trays filled with a 1∶1∶1 mixture of common garden soil, pumice (diameter 1–3 mm) and lava split (2–5 mm) in a climate chamber at the University of Zurich (light: 23°C/at least 220 µmol photons m^–2^ s^−1^/14 h; dark: 8°C/10 h; humidity: 50%). We randomly selected one seed from each of eight fruits per treatment and mother plant (32 seeds per plant). Seeds from the OP, HC and HS pollination treatment were sown (1 seed per 12 mL soil plug in a 288-plug seedling tray). Seeds from the AS treatment were not used, because this treatment had only been included to investigate the importance of pollinators for seed set. The soil was kept moist by adding water twice a day. The position of the trays was randomized twice a week. Seedlings were counted to determine germination success.

### Drought Stress Experiment

When seedlings had produced open cotyledons (20–34 days after sowing), they were transferred individually to 1 L pots and placed randomly on eight trays in the greenhouse (167 seedlings). The substrate mixture and the environmental conditions were the same as in the germination experiment. Seedlings from each pollination treatment were equally assigned to a normal watering and to a drought treatment. Under normal watering each pot received 120 ml of water every fifth day, under the drought treatment only every eleventh day. In a pilot study this level of drought stress had been identified as the level at which seedlings were just before the point of permanent wilting [cf. 38, 39]. After 32 days we recorded the number of surviving seedlings, counted their leaves and measured the length of the longest leaf. Since leaf number and size were strongly correlated (r = 0.79, P<0.001), we only present results for leaf length.

### Data Analysis

The magnitude of inbreeding depression (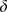) was calculated as:
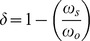
where and are the mean values of progeny fitness from the HS and HC treatments, respectively [40].

We used linear mixed models based on restricted maximum likelihood (REML) estimation and Wald statistics with approximate F-tests to analyse variation in the response variables seed number per fruit, seed mass and leaf length. Population size (i.e. log[number of flowering plants per population]), pollination treatment and their interaction were treated as fixed effects and population identity, mother plant identity and their interactions with pollination treatment as random effects. In the model for the response variable seed number the effect of the pollination treatment was partitioned into three orthogonal contrasts [41], [42]: autonomous selfing (AS) vs. the average of open-pollination (OP) and the two hand pollination treatments (HS and HC), OP vs. the average of HS and HC, and HS vs. HC (Table 1). For the other response variables the first contrast was not possible, because the seeds from the AS treatment were discarded (Table 2). The distribution of residuals was visually inspected to check whether it met assumptions of normality and homoscedasticity [42]. A generalized linear mixed model with binomial error distribution and a logit link [42] was fitted to analyse the effects of population size, pollination treatment contrasts and the interactions between population size and treatment contrasts on the proportion of germinated seeds.

**Table 1:** Linear mixed model analysis of the effects of population size and pollination treatments on the number of seeds per fruit.

Source of variation	Survival			Leaf length		
	Df	*F*	*P*	Df	*F*	*P*
Pollination treatment						
Open vs. hand	1, 159.7	3.07	0.082	1, 120.2	0.15	0.726
Hand-selfed vs. hand-crossed	1, 157.2	22.73	**<0.001**	1, 121.1	5.37	**0.022**
Drought stress	1, 136.2	30.16	**<0.001**	1, 121.9	287.83	**<0.001**
Pollination treatment × drought stress						
Open vs. hand × drought stress	1, 117.0	0.63	0.427	1, 113.2	3.33	0.071
Hand-selfed vs. hand-crossed × drought stress	1, 120.2	4.16	**0.044**	1, 116.3	0.06	0.805

Pollination treatment was partitioned into three orthogonal contrasts: Autonomously selfed vs. other (open or hand pollinated), open pollinated vs. hand pollinated, and hand-selfed vs. hand-crossed.

**Table 2:** Linear mixed model analysis of the effects of population size and pollination treatments on seed mass.

Source of variation	Df	*F*	*P*
Population size	1, 5.5	6.53	**0.046**
Pollination treatment			
Open vs. hand	1, 6.6	8.77	**0.022**
Hand-selfed vs. hand-crossed	1, 6.5	0.36	0.568
Pollination treatment × population size			
Open vs. hand × population size	1, 6.3	0.41	0.546
Hand-selfed vs. hand-crossed × population size	1, 6.2	0.96	0.364

Pollination treatment was partitioned into two orthogonal contrasts: Open pollinated vs. hand pollinated, and hand-selfed vs. hand-crossed.

To investigate the effect of drought stress on leaf length and seedling survivorship (i.e. proportion of seedlings that survived until the end of the experiment), we used mixed model analyses with drought stress, pollination treatment and their interaction treated as fixed effects and tray and mother plant identity as random effects (Table 3). Population size and identity were omitted to avoid over-fitting these models. Leaf length was analysed using linear mixed models, while for the analysis of seedling survival a generalized mixed model with binomial error distribution and a complementary log-log link was used [43]. Classical general and generalized linear model analyses of the data yielded qualitatively identical results, highlighting the robustness of our findings.

**Table 3:** Mixed model analysis of the effects of pollination treatments and drought stress on seedling survival and length of the longest leaf.

		Survival			Leaf length		
Source of variation		Df	*F*	*P*	Df	*F*	*P*
Pollination treatment							
Open vs. hand		1, 159.7	3.07	0.082	1, 120.2	0.15	0.726
Hand-selfed vs. hand-crossed		1, 157.2	22.73	**<0.001**	1, 121.1	5.37	**0.022**
Drought stress		1, 136.2	30.16	**<0.001**	1, 121.9	287.83	**<0.001**
Pollination treatment × drought stress							
Open vs. hand × drought stress		1, 117.0	0.63	0.427	1, 113.2	3.33	0.071
Hand-selfed vs. hand-crossed × drought stress		1, 120.2	4.16	**0.044**	1, 116.3	0.06	0.805

Pollination treatment was partitioned into two orthogonal contrasts: Open pollination vs. hand pollination and hand-selfed vs. hand-crossed. Variation in leaf length was analysed using a linear mixed model, while a generalized mixed model with a binomial error structure and a complementary log-log link was fitted to analyse the proportion of seedlings that survived until the end of the experiment (see Materials and methods).

All statistical analyses were carried out using the statistical software package GenStat Version 14.1 [44]. Arithmetic means ±1 SE are reported.

## Results

### Pollination Effects on Seed Set, Seed Mass and Germination

**Figure 2:**
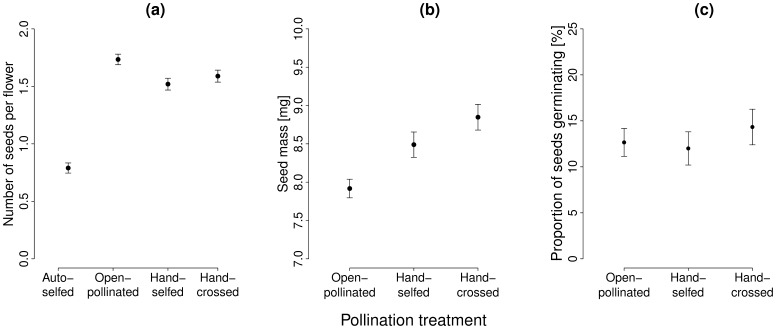
Effects of pollination treatments on seed set, seed mass and germination. Mean values (±1 standard error) of (a) the number of seeds per flower, (b) seed mass and (c) the proportion of germinated seeds of open pollinated, hand-selfed and hand-crossed flowers of *E. wildpretii*. To estimate levels of autonomous selfing, pollinators were excluded by bagging of the flowers. Selfed flowers were hand-pollinated with pollen from the same plant, and crossed flowers with pollen from the most distant plant within a population. The standard error was calculated based on the residual variation of the statistical model.

Flowers that were shielded from pollinators and left to autonomously self-pollinate produced only 55% of the number of seeds produced by open-pollinated flowers (Table 1; Fig. 2a). Open-pollinated flowers produced more (Table 1; Fig. 2a), but smaller seeds (Table 2; Fig. 2b) than hand-pollinated flowers. Germination of open-pollinated flowers was not significantly different from that of hand-pollinated flowers (Fig. 2c). Overall, the seed set, seed mass and germination rate of seedlings of hand-selfed flowers was similar to that of hand-crossed flowers (Table 1,2; Fig. 2a,b,c), resulting in low inbreeding depression (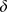 = 4.4% for seed set, 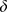 = 4.1% for seed mass and 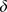 = 7.3% for germination).

**Figure 3:**
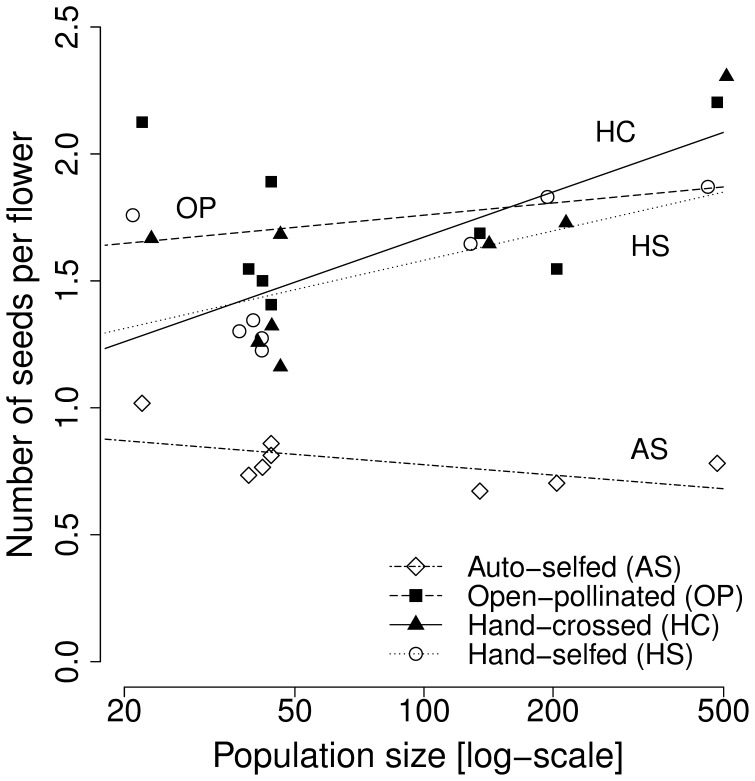
Relationship between seed set and population size (i.e. the log[number of flowering plants per population]) for different pollination treatments. Number of seeds produced by autonomously-selfed (AS), open-pollinated (OP), hand-crossed (HC) and hand-selfed (HS) flowers in populations.

The size of a population tended to affected seed set (*F*_1,6.0_ = 3.98, *P* = 0.093) and its effect varied depending on pollination treatment (pollination treatment × population size interaction: (*F*_3,18.0_ = 57.57, *P* = <0.001; Fig. 3). Seed set of autonomously selfed flowers tended to be lower in large populations, while seed set of open-pollinated and hand-selfed, but particularly that of hand-crossed flowers increased with population size (45% increase in the largest compared to the smallest population). In contrast, there was no significant effect of population size on seed mass or germination, and no interaction with pollination treatment (all *P*>0.1).

### Pollination Effects on Offspring Performance Under Drought Stress

**Figure 4:**
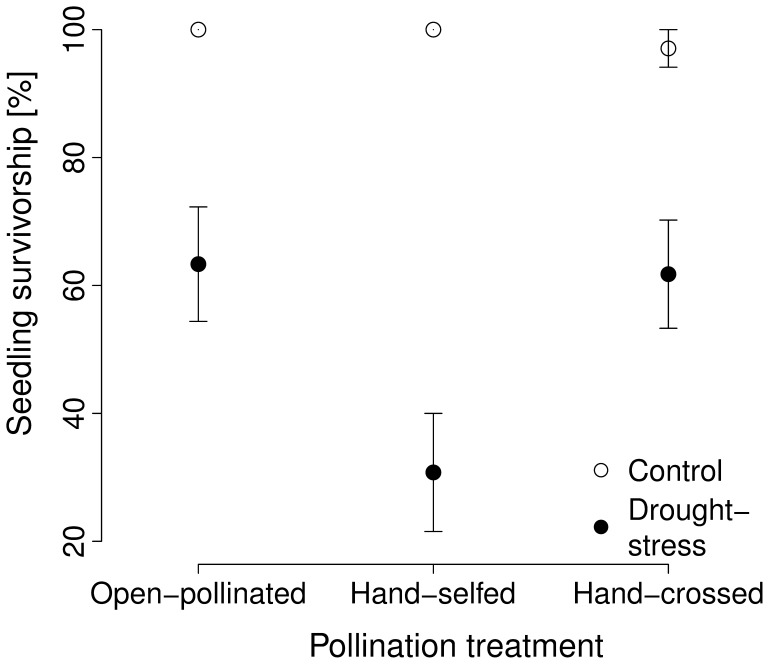
Effect of drought stress on the survivorship of open-pollinated, hand-selfed, and hand-crossed seedlings. Seedlings in the drought stress treatment received 120 ml of water every 11th day, while seedlings in the control treatment (ample watering) received 120 ml every 5th day. The standard error was calculated based on the residual variation of the statistical model.

Overall survivorship was lower for seedlings produced from selfed (64.0±6.9%) than for seedlings produced from hand-crossed seeds (79.4±4.9%) (Fig. 4; Table 3). However, the seedlings from selfed seeds were larger and had longer leaves (8.4±0.3 cm) than those from hand-crossed seeds (7.9±0.2 cm; Table 3). Drought-stressed seedlings had lower survivorship (53.3±5.3% vs. 98.7±0.1%; Table 3; Fig. 4) and shorter leaves (6.0±0.1 cm vs. 9.4±0.1 cm; Table 3) than normally watered seedlings. There was strong inbreeding depression in seedling survivorship under drought stress (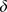 = 50.2%), but not under normal watering (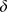 = –3.0%; Fig. 4; Table 3: significant interaction between watering treatment and the selfed vs. hand-crossed contrast). No significant inbreeding depression was found for leaf length (Table 3), neither under drought stress (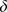 = 2.0%) nor normal watering (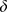 = –0.3%).

## Discussion

### Inbreeding Depression

Selfing resulted in inbreeding depression at several life-history stages of *E. wildpretii*. However, strong inbreeding depression of 50% was only found for the survivorship of seedlings under drought stress, while under ample water availability there was no such adverse effect of selfing on progeny survivorship. Inbreeding effects on seed mass and seed germination varied among populations or maternal plants.

Inbreeding depression may be more evident in late life history stages due to the accumulation of mildly deleterious mutations [45]. In *E. wildpretii*, selfing did not negatively affect seedling performance as measured by seedling survival and leaf length under ample watering. This indicates that under benign environmental conditions late acting inbreeding depression was not expressed or, alternatively, that it is expressed in traits even later in the life cycle not measured in this study, such as reproductive traits [46]. However, under drought stress, seedling survivorship was dramatically lower in selfed than in hand-crossed seedlings. Thus, late-acting inbreeding depression was only expressed under stressful conditions. As in *Lychnis flos-cuculi* [21], only survivorship, but not seedling performance showed this strong interaction between inbreeding depression and drought stress.

Several studies have found that inbreeding depression is more severe under stressful conditions [16], [23], [47], suggesting that recessive alleles become more deleterious under stressful conditions. However, this is not a universal pattern [22], [23]. Recently, it has been suggested that the interactions between inbreeding and the environment may be more complex than previously thought [25], [48]. A recent meta-analysis found no evidence for stronger inbreeding under field than under glasshouse or garden conditions [24]. Variation in population size has been hypothesized to shape the impacts of environmental stress on inbreeding in patchy populations [26], although such interactive effects are currently not well understood [23]. In *E. wildpretii* drought stress clearly increased inbreeding depression. However, we found little evidence for interactive effects of drought stress and population size on the level of expressed inbreeding depression.

Interactions of stress and inbreeding may be a result of direct effects of inbreeding on loci involved in stress susceptibility through increased homozygosity of loci containing stress-sensitive alleles [49], and from indirect effects of enlarged differences in overall vigour between inbreeding groups as a consequence of costs of resistance mechanisms induced under stressful conditions [20], [50].

In the present study we focused on inbreeding effects comparing selfing with within-population hand-outcrossing. Crossings between plants from different populations may further improve fitness compared to selfed offspring through heterosis [14], [15], [22], [38], [51], which may mitigate some of the adverse effects of inbreeding in populations [52], but can also have adverse effects through outbreeding depression [53].

### Effects of Population Size

Our finding that the seed set of autonomously selfed flowers was only slightly more than half of that of open-pollinated flowers demonstrates that, although *E.wildpretii* is capable of producing seeds through autogamy or wind pollination, animal pollinators strongly increase the seed set in this protandrous species. Animal-pollinated plants may be particularly negatively affected by small population size as a consequence of reduced pollination and reproduction (e.g. [54]). Such Allee effects may include quantitative pollination limitation through reduced pollinator attraction [55] and qualitative pollen limitation through increased mating among close relatives and decreased pollen competition [11], [13], [19], [56]. However, the results of studies on the effects of small population size on plant fitness that are mediated by pollinators have been inconsistent (reviewed in [17], [57], [58]). For a long-lived monocarp such as *E. wildpretii* such effects could be particularly severe, as the number of flowering plants is usually less than 10% of total population size, with often only few and widely spaced flowering individuals in a population. Indeed, seed set produced by open-pollinated flowers tended to increase with population size. Together with our finding that seed set of manually hand-crossed flowers also increased with population size this suggests that genetic variability between plants was higher in large than in small populations, resulting in lower bi-parental inbreeding depression [16], [59]. Fitness measures at later life-history stages might also be expected to be affected, as they have a higher probability of accumulating deleterious alleles [18], [60]–[63].

### Conclusions

Our results demonstrate different levels of inbreeding depression expressed at different life-history stages of *E. wildpretii*. Moreover, the reduced positive effects of hand-outcrossing in small populations of *E. wildpretii* indicate increased biparental inbreeding resulting in inbreeding depression in these populations. However, severe inbreeding depression after selfing was only expressed in offspring survivorship under drought stress, emphasizing the importance of studying inbreeding effects under different environmental conditions. Drought stress is predicted to increase as a consequence of an increase in temperature and a decrease in precipitation, resulting in longer and more frequent drought events in the Canary Islands ([27], [28] and references therein). Moreover, there is evidence that introduced honeybees cause higher levels of selfing in *E. wildpretii* compared with native pollinators [31], as they typically move more among flowers within plants but often less among different plants than native pollinators do [64], [65]. Thus, effects of predicted climate change in combination with increased selfing by honeybees may pose a special threat to successful seedling recruitment in *E. wildpretii*.
